# Three new species of caddisflies (Trichoptera, Hydroptilidae, Polycentropodidae, Leptoceridae) from Khon Phapheng Waterfall, the Mekong River, Laos

**DOI:** 10.3897/zookeys.1055.66536

**Published:** 2021-08-12

**Authors:** Penkhae Thamsenanupap, Hans Malicky, Chanda Vongsombath, Pongsak Laudee

**Affiliations:** 1 Faculty of Environment and Resource Studies, Mahasarakham University, Mahasarakham Province, Thailand Mahasarakham University Mahasarakham Thailand; 2 Sonnengasse 13, A-3293 Lunz am See, Austria Unaffiliated Lunz am See Austria; 3 Faculty of Environmental Sciences, National University of Laos, Dong Dok Campus, Vientiane, PDR, Laos National University of Laos Vientiane Laos; 4 Faculty of Innovative Agriculture and Fishery Establishment Project, Prince of Songkla University, Surat Thani Campus, Muang District, Surat Thani Province, Thailand Prince of Songkla University Surat Thani Thailand

**Keywords:** Aquatic insects, biodiversity, Oriental Region, taxonomy

## Abstract

The Mekong River is a hotspot area for freshwater biodiversity, but caddisfly diversity is largely understudied. Three new species of caddisflies from three different families are described and illustrated from Khon Phapheng Waterfall, the Mekong River, Laos; *Orthotrichiachoengthongi* Malicky & Laudee, **sp. nov.** (Hydroptilidae), *Pseudoneureclipsiskhonphaphengensis* Malicky & Thamsenanupap, **sp. nov.** (Polycentropodidae), and *Setodeskarrilai* Malicky & Laudee, **sp. nov.***Orthotrichiachoengthongi* Malicky & Laudee, **sp. nov.** can be differentiated from the most similar *Orthotrichiatriton*Malicky 2008 by the specific shape of segment X which is long and tubular, pointed apically and curved inward then immediately upward in dorsal view. *Pseudoneureclipsiskhonphaphengensis* Malicky & Thamsenanupap, **sp. nov.** differs from the similar *P.kaineus* Malicky & Bunlue in Malicky et al. 2004 by the shape of the inferior appendages that are usually broad, almost circular in lateral view. *Setodeskarrilai* Malicky & Laudee, **sp. nov.** is mainly different to *S.omphale* Malicky & Changthong in Malicky et al. 2004 by the distal part of segment X which has a brush-like process.

## Introduction

The Mekong River is one of the main rivers in Southeast Asia, flowing from the Tibetan Plateau through several countries to the South China Sea. A large human population in the Southeast Asian region depends on it as a source for subsistence (Campbell 2009; Mekong River Commission 2020). The ecological system in the Mekong River is also varied as it runs from the Tibetan Plateau through to the South China Sea, and the lower Mekong basin is a hotspot area for aquatic biodiversity (Campbell 2009). At the place where the Mekong River zigzags from the Korat Plateau, which is in the northeastern Thailand, through the Tonle Sab basin, a broad valley to the east of the Khorat Plateau, the river level drops several meters over many waterfalls, such as Li Phi fall and Khon Phapheng waterfall (Campbell 2009).

Knowledge on species diversity of Trichoptera in Laos is very limited. Approximately two hundred Laotian caddisfly species have been recorded (Malicky 2010). Most of the studies focused on caddisfly fauna were conducted in northern, central, and southern Laos encompassing only a few localities in each area, such as Bokeo Province, Luang Prabang Province, Kham Muan Province and Pakse Province (Malicky and Chantaramongkol 2006; Laudee and Malicky 2017; Malicky and Laudee 2019; Laudee et al. 2020). Considering the under-investigated sites in this country, there are probably many species of caddisflies that have not yet been recorded. The three new species of Trichoptera described in this article are from the genera *Orthotrichia* Eaton, *Pseudoneureclipsis* Ulmer, and *Setodes* Rambur. The genus *Orthotrichia*, has not been reported from Laos previously. So far, there are only six Laotian species in the genus *Pseudoneureclipsis*, namely *P.usia* Malicky & Chantaramongkol, 1993, *P.amulius* Malicky, 1997, *P.ramosa* Ulmer, 1913, *P.kaineus* Malicky & Bunlue in Malicky et al. 2004, *Pseudoneureclipsisarael* Malicky & Laudee, 2017, *Pseudoneureclipsishamabiel* Malicky & Laudee, 2017 (Malicky 2010; Laudee and Malicky 2017; Malicky and Laudee 2017). In the genus *Setodes*, twenty-one species have been reported from Laos so far (Malicky 2010; Malicky and Laudee 2017; Malicky and Laudee 2019) by various authors. In this count, nine species were described by Malicky and Chantaramongkol (2006), namely *Setodesthoneti*, *S.horatius*, *S.herakleidos*, *S.meriones*, *S.minotauros*, *S.ischys*, *S.lausus*, *S.larva* and *S.leukothea*. Recently, eight species of *Setodes* were described from Laos, *Setodesmarianu* Malicky & Laudee, 2017 and seven new species by Malicky and Laudee (2019), namely *S.dubiel*, *S.tamiel*, *S.nithael*, *S.paniel*, *S.nanael*, *S.lezalel*, and *S.sachiel*. In addition, four species were recorded from Laos including *S.tarpaka* Gordon & Schmid, 1987, *S.brevicaudatus* Yang & Morse, 1989, *S.cheni* Yang & Morse, 2000, and *S.metis* Malicky & Thapanya in Malicky and Chantaramongkol 2006.

The aim of this research study was to describe new species of Trichoptera from the falls in the Mekong River, and thus to provide a valuable contribution to the knowledge on South-East Asian caddisfly diversity.

## Materials and methods

Adult stage caddisfly specimens were collected with a UV pan light trap (12 V, 10 W) set beside Khon Phapheng waterfall, Mekong River, at night. The caddisfly specimens were preserved in 70% ethanol. For identifying to species level, the male genitalia of the caddisflies were observed under a stereomicroscope. The male genitalia were dissected and the muscle tissues macerated by heating in 10% KOH at 60 °C for 30–60 minutes then soaked in a detergent solution before being transferred back to 70% ethanol. The genitalia of caddisflies were identified to species level after Malicky (2010). For the new species of caddisflies, drawings of the male genitalia were made in pencil using a compound microscope equipped with a drawing tube, and the drawings were used to produce the final vector graphics in Adobe Illustrator software.

Holotypes are preserved and stored in the Princess Maha Chakri Sirindhorn Natural History Museum, Prince of Songkla University, Hat Yai Campus, Hat Yai district, Songkhla province, Thailand (**PSUNHM**). Some paratypes are deposited in the collection of Hans Malicky (**CHM**), the Clemson University Arthropod Collection (**CUAC**), and the National Museum, Prague, Czech Republic (**NMPC**). Terminology for genitalia structures for different genera follows Wells (1979) and Marshall (1979) for the genus *Orthotrichia*, Oláh and Johanson 2010 for the genus *Pseudoneureclipsis*, and Laudee and Malicky 2016 for the genus *Setodes*.

## Systematics

### Orthotrichia choengthongi

Taxon classificationAnimaliaTrichopteraHydroptilidae

Malicky & Laudee sp. nov.

F0890D04-361E-5527-A131-72102E56F397

http://zoobank.org/056A2710-8A8C-4439-BE95-7E45782B9C07

Figure 1

#### Type material.

***Holotype.* Male. Laos: Pakse Province**: Champasak, Khonphapheng waterfall, Mekong River, 13°57'30"N, 105°59'14"E, elev. 64 m, 8.iv.2019, Pongsak Laudee. (PSUNHM). ***Paratypes***: same data as the holotype; **Laos, Pakse Province**: Champasak, Don Khon, Mekong river, 13°57'45"N, 105°55'07"E, elev. 84 m, 8.iv.2019, Pongsak Laudee. 11 males: 3 males (PSUNHM), 3 males (CHM), 2 males (NMPC), 3 males (CUAC).

#### Diagnosis.

The new species is similar to *Orthotrichiatriton* Malicky, 2008 from Borneo in the general shape of the male genitalia. However, it can be differentiated in that the shape of tergum X of *Orthotrichiachoengthongi* Malicky & Laudee, sp. nov. is long and tubular, pointed apically and curved inwards then abruptly upwards in dorsal view; however, is stout basally then curved slightly downward in *O.triton*. Moreover, the united inferior appendages of the new species are collectively trilobed; but bilobed in *O.triton*.

#### Description.

Adult, male, length of each male forewing 1.6.–2.0 mm (n = 11); color in alcohol of head, thorax, forewings, abdomen, and legs light brown.

**Figure 1.**
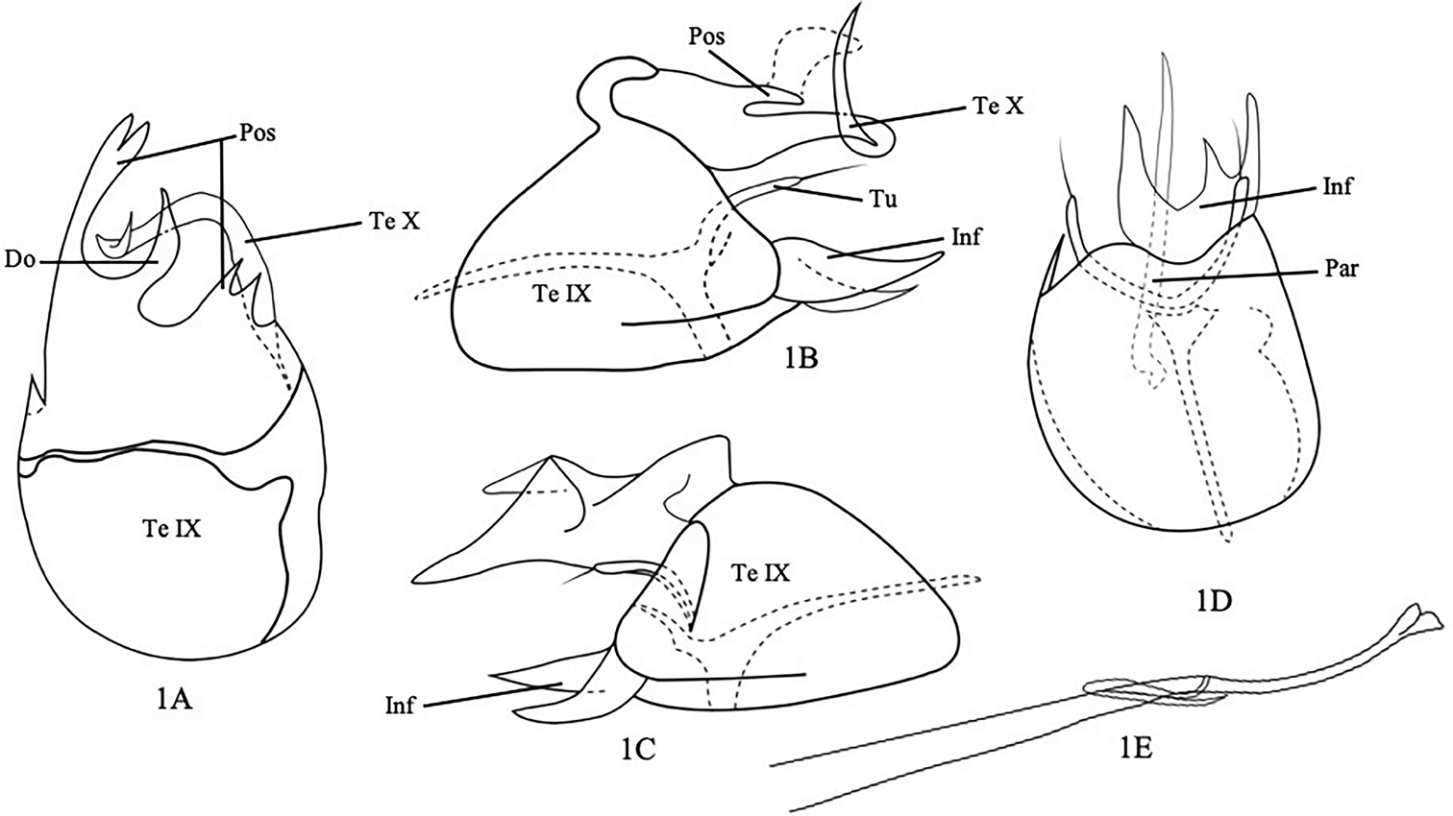
*Orthotrichiachoengthongi*, sp. nov. Male genitalia **A** tergum IX and tergum X, dorsal **B** terga IX and X and inferior appendages, left lateral **C** terga IX and X and inferior appendages, right lateral **D** inferior appendages and paramere, ventral **E** phallus, left lateral. Abbreviations: Te IX = tergum IX, Pos = Posterolateral process (paired), Do = Doral spine, Te X = tergum X, Inf = united inferior appendages, Tu = Tubular processes, Par = Paramere.

Male genitalia extremely asymmetric (Fig. 1A–E). Tergum IX broadly rounded in dorsal and ventral views (Fig. 1A, D); somewhat triangle, anterior margin rounded, with hooked posterodorsal process in left lateral view (Fig. 1B); somewhat triangle in right lateral view (Fig. 1C). Posterolateral processes of tergum IX asymmetrical; left process shorter, bilobed, each lobe pointed apically; right process longer, tubular, bifurcate apically with acute apices in dorsal view (Fig. 1A); in left lateral view, directed caudad, short, pointed apically (Fig. 1B); in right lateral view (Fig. 1C), somewhat triangular, short. Dorsal spine of tergum IX lanceolate, curved upward, pointed apically in dorsal view (Fig. 1A). Tergum X long tubular, pointed apically, curved mesad then abruptly upward in dorsal view (Fig. 1A); shaped nearly as isosceles triangle, pointed apically in right lateral view. United inferior appendages trilobed, asymmetrical, each lobe shaped as isosceles triangle and acute apically, with middle lobe smallest and closer to right lobe in ventral view, left lobe thicker than right one in lateral view (Fig. 1B, C). Pair of tubular processes of inferior appendages long, slender, each with long seta apically (Fig. 1B–D). Paramere spine long, acute apically in ventral view (Fig. 1D). Phallus, long, enlarged and bilobed apically, with titillator encircling segment 1 time tightly against it near the middle (Fig. 1E).

#### Etymology.

The species epithet honors Asst. Prof. Dr Suchart Choengthong, Prince of Songkla University, Surat Thani Campus.

### Pseudoneureclipsis khonphaphengensis

Taxon classificationAnimaliaTrichopteraPolycentropodidae

Malicky & Thamsenanupap sp. nov.

38177D87-4B4D-5F81-996C-DFA479D8959A

http://zoobank.org/CDDB7489-A5C2-4371-B4B8-F3CDE6CA538C

Figure 2

#### Type material.

***Holotype*. Male. Laos: Pakse Province**: Champasak, Khonphapheng waterfall, Mekong river, 13°57'30"N, 105°59'14"E, elev. 64 m, 8.iv.2019, Pongsak Laudee. (PSUNHM). ***Paratypes***: same data as the holotype; **Laos: Vientiane province**: Nam Lik river, 18°31'29"N, 102°31'19"E, elev. 180 m, 8.iv.2019, Pongsak Laudee. 9 males: 2 males (PSUNHM), 3 males (CHM), 2 males (NMPC), 2 males (CUAC).

#### Diagnosis.

The male genitalia of *P.khonphaphengensis* sp. nov. are similar to those of *P.kaineus* Malicky & Bunlue in Malicky et al. 2004 from Thailand. However, *P.khonphaphengensis* sp. nov. is strikingly different from *P.kaineus* and the other species of the genus by the usually broad, almost circular inferior appendages with 1.5 × longer than its width in lateral view, while the inferior appendages of *P.kaineus* are oval and 3.2 × longer than its width in lateral view. In addition, the phallus of *P.khonphaphengensis* sp. nov. bearing the hooked phallic sclerite which are lacking in *P.kaineus*.

#### Description.

Adult, male, length of each male forewing 4.0–4.5 mm (n = 9); general body color in alcohol yellow brown.

**Figure 2.**
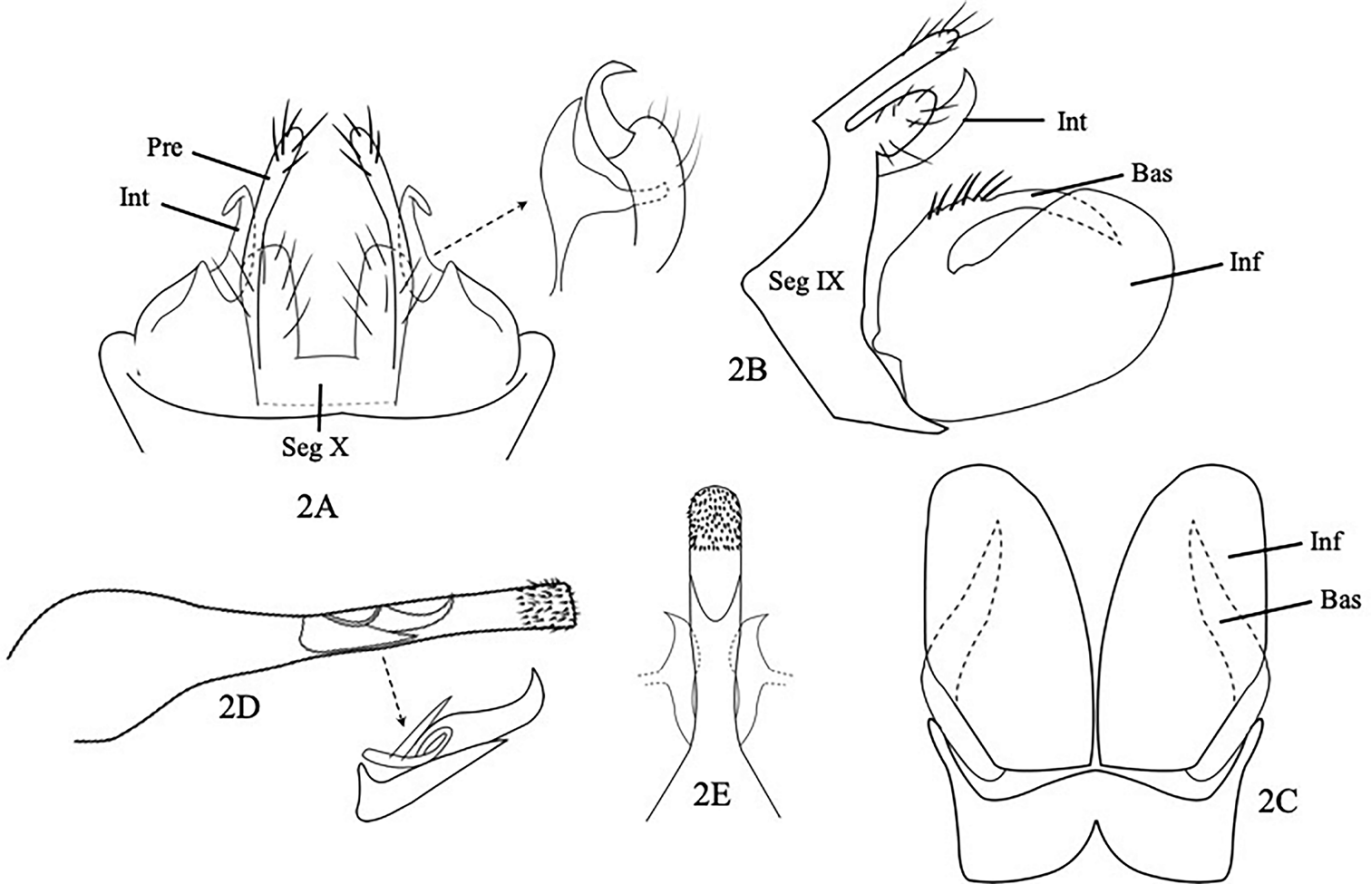
*Pseudoneureclipsiskhonphaphengensis*, sp. nov. Male genitalia **A** segment X, preanal appendages and intermediate appendages, dorsal **B** segments IX and X and inferior appendages, left lateral **C** inferior appendages, ventral **D** phallus, left lateral; the arrow shows hooked phallic sclerite, ventral **E** phallus, dorsal. Abbreviations: Seg IX = segment IX, Seg X = segment X, Pre = preanal appendages, Int = Intermediate appendage, Inf = inferior appendage (paired), Bas = basodorsal process of inferior appendage.

Male genitalia as in Figure 2A–E. Segment IX narrow in lateral view, with anterior margin produced forward in ca. 100° angle. (Fig. 2B). Segment X bilobed, blunt apically in dorsal view; finger like with setae in lateral view. Preanal appendages, long, slender with setae, rounded apically in dorsal view and lateral view (Fig. 2A, B). Intermediate appendages hooked and turned dorsally then bend outward immediately in dorsal view (Fig. 2A); in lateral view hooked, slightly curved upward, pointed apically (Fig. 2B). Inferior appendages strikingly broad and rounded, total length < 2 × of its subapical width in lateral view (Fig. 2B); slender oviform, total length < 2 × of its width in ventral view (Fig. 2C). Basodorsal processes of inferior appendages curved with strong setae dorsomedially, pointed apically in lateral view (Fig. 2B); in ventral view, horn like, originated basodorsally, curved inward, each with tapering apex (Fig. 2C). Phallus, long, cylindrical, bulbous basally, straight laterally with hooked phallic sclerite intermediately, with numerous subapical spines in lateral view and dorsal view (Fig. 2D, E).

#### Etymology.

The species is named for the type locality, Khon Phapheng waterfall.

### Setodes karrilai

Taxon classificationAnimaliaTrichopteraLeptoceridae

Laudee & Malicky sp. nov.

E391C5D4-BD90-511E-9478-384690204BD3

http://zoobank.org/7CE4F44E-4315-4313-A7B6-C276EC4F46C0

Figure 3

#### Type material.

***Holotype*. Male. Laos: Pakse Province**: Champasak, Khonphapheng waterfalls, Mekong river, 13°57'30"N, 105°59'14"E, elev. 64 m, 8.iv.2019, Pongsak Laudee. (PSUNHM). ***Paratypes***: same data as the holotype; **Laos: Vientiane province**: Nam Lik river, 18°31'29"N, 102°31'19"E, elev. 180 m, 8.iv.2019, Pongsak Laudee; **Laos, Pakse province**: Champasak, Don Khon, Mekong river, 13°57'45"N, 105°55'07"E, elev. 84 m, 8.iv.2019, Pongsak Laudee; **Laos: Vientiane province**: Nam Ngum river, 18°31'29"N, 102°31'37"E, elev. 180 m, 8.iv.2019, Pongsak Laudee. 31 males: 10 males (PSUNHM), 8 males (CHM), 5 males (NMPC), 5 males (CUAC).

#### Diagnosis.

The male genitalia of *S.karrilai* sp. nov. are similar to those of *S.omphale* Malicky & Changthong in Malicky et al. 2004 from Thailand. Both species share similar characteristics of five lobes of each inferior appendage. However, the most prominent difference is the structure of segment X, i.e., the distal part of segment X in *S.karrilai* sp. nov. has a brush-like process, visible in lateral and dorsal views, which is lacking in *S.omphale*. In addition, the phallus of *S.karrilai* sp. nov. has a pair of very long, thin, pointed parameres with distal 1/3 bent and twisted meso-upward; whereas the parameres of *S.omphale* are curved downward.

#### Description.

Length of each male forewing 6.5–7.0 mm (n = 18); color in alcohol of head, thorax, forewings, and abdomen yellow; femora and tibiae brown or dark brown; wings clear and transparent, yellowish with brown veins.

**Figure 3.**
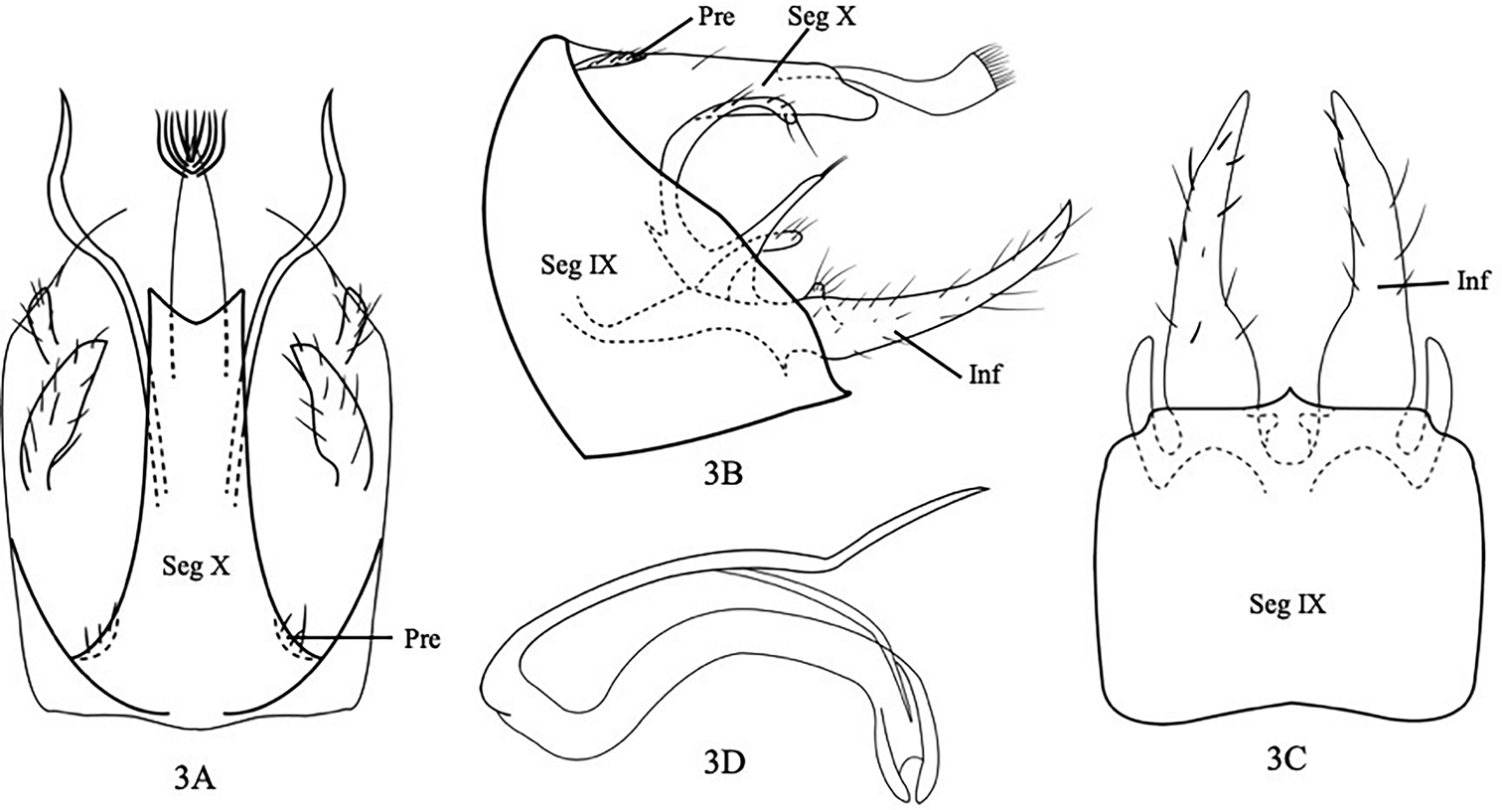
*Setodeskarrilai*, sp. nov. Male genitalia **A** segment X, preanal appendages, dorsal **B** segments IX and X, preanal appendages, inferior appendages, left lateral **C** segment IX, inferior appendages, ventral **D** phallus, left lateral. Abbreviations: Seg IX = segment IX, Pre = preanal appendage (paired), Inf = inferior appendage (paired).

Male genitalia as in Figure 3. Segment IX somewhat triangular with long sinuous edges posteriorly, convex anteriorly in lateral view (Fig. 3B); square in ventral view (Fig. 3C). Segment X long, slender, divided into basal segment and apical segment (Fig. 3A, B); basal segment mostly tubular with shallow and broad excision apically, apical segment slender, tubular with brush of straight hairs at posterior end in dorsal view (Fig. 3A); in lateral view, basal segment tubular, apical segment thicker apically and with apical brush (Fig. 3B). Preanal appendages very small, leaf-like with scattered setae (Fig. 3A, B). Inferior appendages each five-lobed (Fig. 3B); dorsal lobe long, tubular and erect basally then curved caudad, two finger-like mesal lobes tubular and with upper mesal lobe longer than lower mesal lobe, basoventral lobe small and knot-like, ventral lobe very long and sword-like with scattered setae in lateral view (Fig. 3B). Phallus long, slender, bent downwards with pair of very long, thin, and pointed parameres with distal 1/3 bent and twisted meso-upward in lateral view (Fig. 3D).

#### Etymology.

The species epithet honors Assoc. Prof. Dr Seppo Karrila, Faculty of Science and Industrial Technology, Prince of Songkla University, Surat Thani Campus.

## Discussion

**Figure 4.**
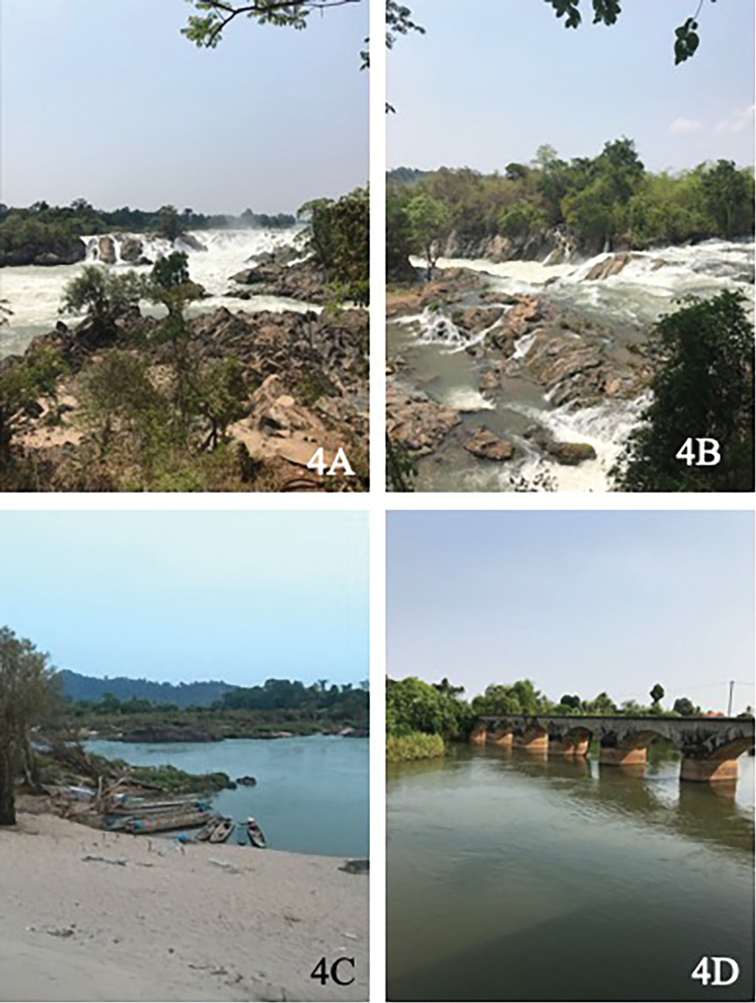
Study sites from Mekong **A, B** Khon Phapheng Waterfall, Paksong, Pakse Province **C, D** Don Khon, Paksong, Pakse Province.

Three new species of caddisflies were described from Khon Phapheng waterfall, where the whole Mekong River drops 30 meters over the waterfalls. These species are *Orthotrichiachoengthongi* sp. nov., *Pseudoneureclipsiskhonphaphengensis* sp. nov., and *Setodeskarrilai* sp. nov. The three new species are potamon species that live in large rivers. The substrate is dominated by bedrocks, boulders, and sand (Fig. 4A–D). There is no previously reported species of *Orthotrichia* in Laos. However, more than 50 species of the genus have been reported from adjacent countries (Malicky 2010), thus we expect additional new findings of this genus in Laos, in further surveys. Most of *Orthotrichia* spp. in Asia were found from waterfalls and streams with large-sized lithal as the dominant substrate (e.g., boulders, Malicky and Chantaramongkol 2007; Ito and Park 2016), as in the study sites at the Khon Phapheng waterfall. Moreover, Well and Dudgeon 1990 reported that the larvae are often found under rocks in streams. The *Orthotrichia* spp. in South-East Asia show restricted regional distribution, as only 9 of the 54 species are reported to be widely distributed (Malicky and Chantaramongkol 2007; Malicky 2010).

The genus *Pseudoneureclipsis* has been previously reported from Laos, i.e., six species were known; thus including *P.khonphaphengensis* sp. nov. there are now seven species of *Pseudoneureclipsis* recorded from Laos. In addition to the Laotian species, there are three species (*P.romosa*, *P.usia* and *P.kaineus*) with wider distribution in South-East Asia (Malicky 2010). *Pseudoneureclipsisromosa* and *P.kaineus* are potamon species as they were found from the Mekong River (Laudee and Malicky 2017) but Thapanya et al. 2004 reported that *Pseudoneureclipsis* spp. are found also in mountain streams in Doi Suthep-Pui and Doi Inthanon National Parks, Chiang Mai Province, Thailand. The larvae of the genus *Pseudoneureclipsis* live on undersides of stones in shallows of fast flowing water (Dudgeon 1999).

In addition to the description of a new species, for *Setodeskarrilai* sp. nov. we give data on its distribution, as it was collected from several provinces in Laos, not only from the Khon Phapheng waterfall. Thus, it is a widespread species in the Mekong River, which is found from central to southern Laos. The genus *Setodes* is represented by twenty Laotian species, among the more than eighty species that have been reported from South-East Asia (Malicky 2010) and the 285 species reported from the Oriental Biogeographic Region (Morse 2021). The genus *Setodes* inhabits a variety of habitats, such as waterfalls, small streams, and rivers (Malicky and Chantaramongkol 2006; Laudee and Malicky 2016). In addition, *Setodes* larvae have been reported to burrow into sand deposits of riffle areas (Merill and Wiggins 1971).

## Supplementary Material

XML Treatment for Orthotrichia choengthongi

XML Treatment for Pseudoneureclipsis khonphaphengensis

XML Treatment for Setodes karrilai
